# Identification of miR-320 family members as potential diagnostic and prognostic biomarkers in myelodysplastic syndromes

**DOI:** 10.1038/s41598-020-80571-z

**Published:** 2021-01-08

**Authors:** Chengyao Wan, Jing Wen, Xiaolin Liang, Qiongni Xie, Wenqi Wu, Meiqing Wu, Zhenfang Liu

**Affiliations:** Department of Hematology, The First Affiliated Hospital of Guangxi Medical University, No. 6, ShuangYong Road, Nanning, 530021 Guangxi China

**Keywords:** Prognostic markers, Haematological cancer, Gene expression

## Abstract

Myelodysplastic syndromes (MDS) are characterized by ineffective hematopoiesis and the abnormal differentiation of hematopoietic stem cells. An increasing number of researches have demonstrated that microRNAs play crucial roles in the pathogenesis of myelodysplastic syndromes. Herein, we aimed to identify novel potential microRNAs bound up with the diagnosis and prognosis of MDS. MiRNA microarray analysis was used to screen deregulated microRNAs in the bone marrow of MDS patients. qRT-PCR was employed to confirm the microarray results. All members of miR-320 family (miR-320a, miR-320b, miR-320c, miR-320d, and miR-320e) were significantly increased in MDS patients compared to normal control. Although we found no correlation between miR-320 family and most clinical characteristics, high miR-320c and miR-320d expression seemed to be associated with high numbers of bone marrow (BM) blasts and worse karyotype. High expression of all the members of the miR-320 family seemed to be associated with a high prognostic score based on International Prognostic Scoring System (IPSS). The areas under the miR-320 family member ROC curves were 0.9037 (P < 0.0001), 0.7515 (P = 0.0002), 0.9647 (P < 0.0001), 0.8064 (P < 0.0001) and 0.9019 (P < 0.0001). Regarding Kaplan–Meier analysis, high miR-320c and miR-320d expression were related to shorter overall survival (OS). Moreover, multivariate analysis revealed the independent prognostic value of miR-320d for OS in MDS. The expression of miR-320 family members was up-regulated in MDS, and miR-320 family members could serve as candidate diagnostic biomarkers for MDS. High expression of miR-320d was an independent prognostic factor for OS in MDS.

## Introduction

Myelodysplastic syndromes (MDS) are clonal disorders characterized by ineffective hematopoiesis and a high risk of transformation to acute myeloid leukemia (AML)^1^. MDS mainly occurs in adults over the age of 60, and the morbidity increases with age. Despite the use of chemotherapy and supportive treatment for MDS patients, the median survival time of MDS patients after diagnosis is still only 2.5 years^2^, and the natural clinical courses and prognosis are extraordinarily heterogeneous. To date, the only way to cure MDS is allogeneic hematopoietic stem cell transplantation (HSCT). However, few MDS patients can tolerate HSCT due to the old age of patients^3^. Therefore, it is particularly important to identify pertinent molecular targets for the effective treatment of MDS, especially specific biomarkers involved in MDS pathogenesis that are relevant to the diagnosis and prognosis of MDS.

MicroRNAs are endogenous small non-coding RNAs that are completely or incompletely complementary to the 3′-UTR of the target gene to regulate gene expression^4^. An increasing number of researches have indicated that microRNAs might participate in hematopoiesis^5–7^. Furthermore, microRNAs have been demonstrated to be promising diagnostic and prognostic biomarkers and are considered as therapeutic molecular targets in MDS^8^. A variety of studies have indicated that numerous microRNAs such as miR-451a^9^, miR-124^10^, and miR-155^11^, are relevant to the prognosis of MDS and might participate in MDS pathogenesis. Recently, a few studies showed that miR-320 family was able to act as tumor suppressors by targeting CDK6 and inhibiting proliferation of colon cancer cell^12^. On the other hand, miR-320a was reported to promote tumorigenesis via targeting TUSC3 in retinoblastoma^13^. Regarding on hematological malignances, miR-320a could inhibit the malignancy of multiple myeloma by targeting PBX3^14^. However, the role of the miR-320 family in MDS is still unclear. Herein, we investigated the diagnostic and prognostic value of the miR-320 family in MDS patients.

## Results

### MiRNA expression profiles of MDS patients

**Figure 1:**
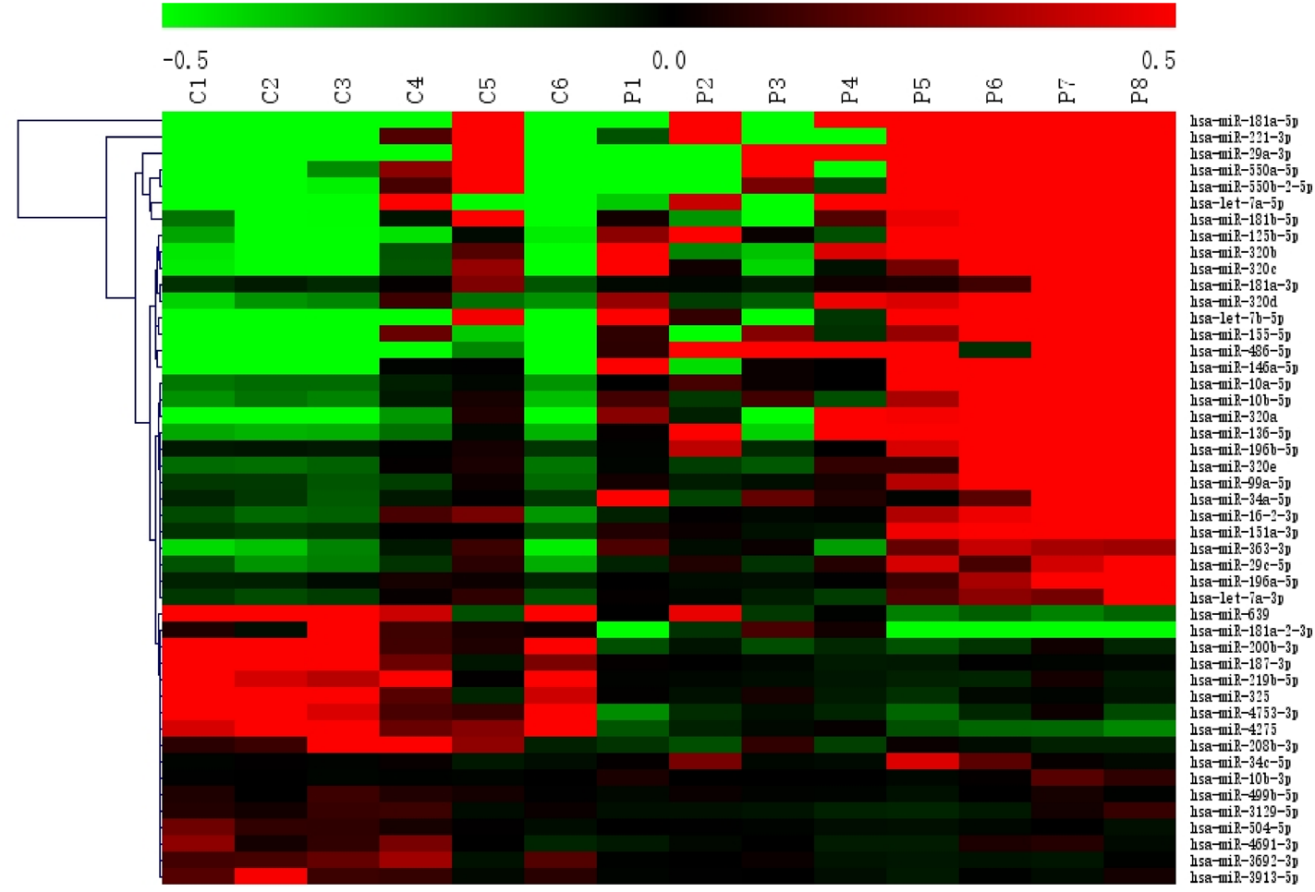
MicroRNA expression patterns in MDS compared with normal bone marrow. Each row represents a miRNA and each column represents a sample. C1–C6 represent controls (n = 6), P1–P4 represent MDS patients with intermediate I (n = 4), P5–P8 represent MDS patients with intermediate II and high-risk (n = 4). Red color indicates up-regulation and green color indicates down-regulation.

Eight MDS patients whose samples were used for microarray analysis were divided into lower risk group (low and intermediate-1 risk, n = 4) and higher risk group (intermediate-2 and high risk, n = 4) based on the IPSS definition. Microarray results showed that the expression of each member of the miR-320 family was close to statistical significance (P < 0.05) and the fold change was higher or slightly lower than 2 between the lower risk group and normal control group. However, the expression of each member of the miR-320 family reached statistical significance (P < 0.05) in the higher risk group, and the fold change was higher than 5 between the higher risk group and normal control (Table 1). Part of microarray results were shown in Fig. 1. Subsequently, miR-320 family members expression was validated by qRT-PCR in a large sample size.

**Table 1:** Identified miR-320 family members expression levels in MDS patients compared with normal control.

MicroRNA	Lower risk/normal		Higher risk/normal	
	Fold-change	P value	Fold-change	P value
miR-320a	2.07	0.064	5.73	**0.001**
miR-320b	1.68	0.098	6.54	**0.006**
miR-320c	1.74	0.158	9.13	**0.019**
miR-320d	2.37	0.058	12.35	**0.014**
miR-320e	1.51	0.343	8.54	**0.006**

Microarray analysis is based on student’s t test. (8 MDS patients vs. 6 controls).

### Validation of miR-320 family members

**Figure 2:**
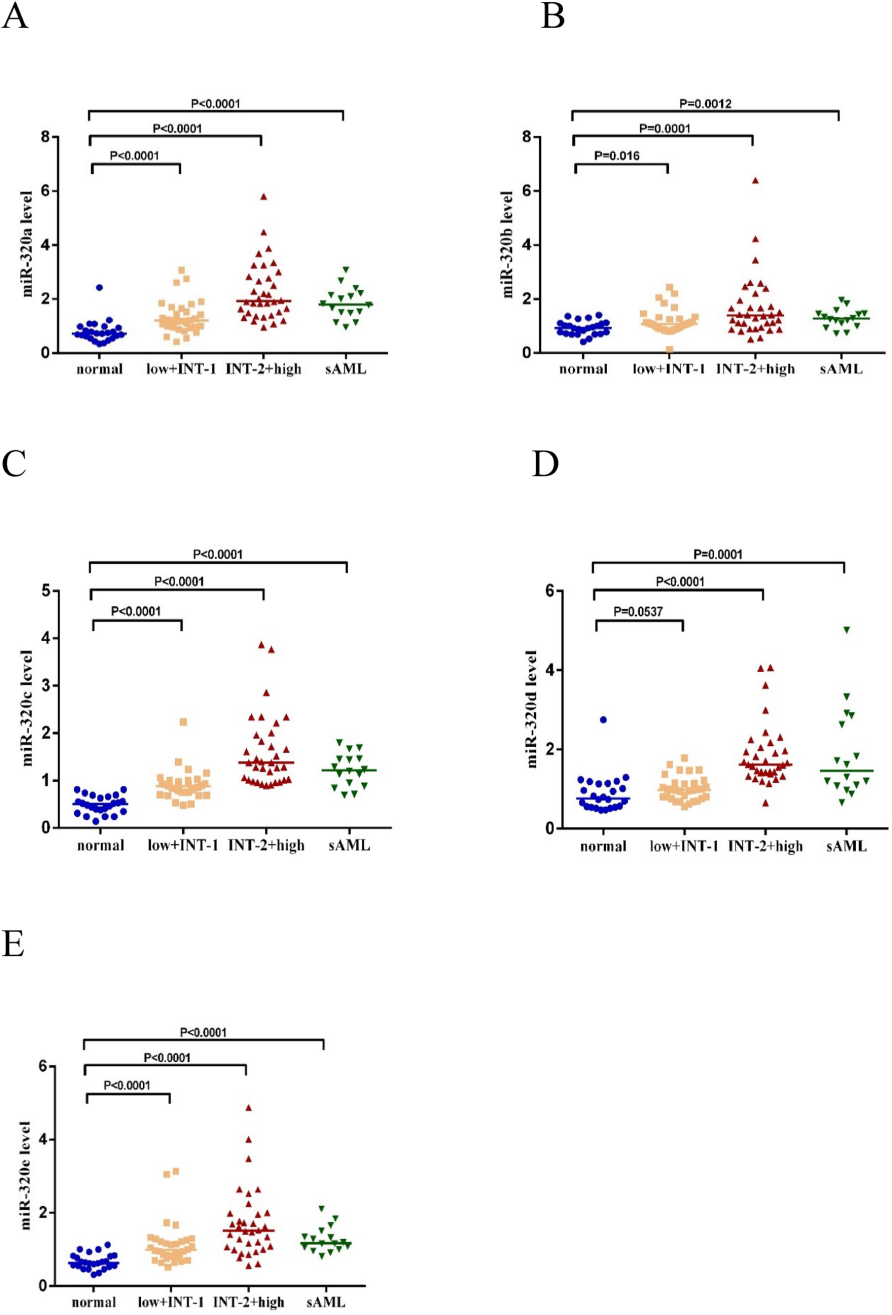
Expression of miR-320 family members in each group. *normal* normal controls (n = 24), *low + INT-1* low risk + intermediate-1 risk (n = 32), *INT-2 + high* intermediate-2 risk + high risk (n = 34), *sAML* secondary acute myeloid leukemia (AML transformed from MDS) (n = 16). Mann–Whitney’s U test was used to compare all the difference of continuous variables between two groups. (**A**) For miR-320a; (**B**) for miR-320b; (**C**) for miR-320c; (**D**) for miR-320d; (**E**) for miR-320e. Bar represents median expression of miR-320 family members.

miR-320 family members expression was validated in BM from 82 MDS patients and 24 normal controls using qRT-PCR. As displayed in Fig. 2, the expression of miR-320 family members was significantly up-regulated in each group of MDS compared with that in normal controls.

### Association of miR-320 family members with clinical characteristics of MDS

To evaluate the clinical implications of miR-320 family member expression, we divided MDS patients into two groups based on the median level of each member of the miR-320 family. The relationship between miR-320 family expression and clinical characteristics was evaluated. As displayed in Table 2, no correlation was found between miR-320 family members and most clinical characteristics. However, high miR-320c and miR-320d expression were associated with a high number of BM blasts (P = 0.003 and P = 0.047), advanced WHO type (P = 0.006 and P = 0.016), and worse karyotype (P < 0.001 and P = 0.024). Additionally, the high expression of all members of the miR-320 family was significantly related to advanced IPSS risk stratification.

**Table 2:** Association of miR-320 family with clinical parameters in MDS patients.

Variables	miR-320a expression			miR-320b expression				
	Low (n = 41)	High (n = 41)	P	Low (n = 41)	High (n = 41)	P		
Sex (male/female)	24/17	27/14	0.494	27/14	24/17	0.494		
Age (years), median (range)	53 (20–78)	48 (19–86)	0.299	48 (19–78)	54 (20–86)	0.703		
WBC (× 10^9^/L), median (range)	2.95 (0.77–145)	3.48 (0.79–69.9)	0.444	3.58 (0.77–145)	3.27 (0.79–10.53)	0.493		
Hemoglobin (g/L), median (range)	64 (35.5–108.3)	60.1 (26.3–103.3)	0.533	64 (35.5–108.3)	60.6 (26.3–103.3)	0.571		
Platelets ( × 10^9^/L), median (range)	51 (1.92–726.8)	46 (51–387)	0.182	46.4 (1.92–726.8)	51 (5.1–496.1)	0.33		
BM blasts (%), median (range)	4.5 (0–90.5)	8.2 (0–61.5)	0.091	4.0 (0–90.5)	7.4 (0–61)	0.177		

**WHO type, n(%)**								
5q^-^/RA/RARS	6 (7.3)	1 (1.2)	0.114	4 (4.9)	3 (3.7)	0.081		
RCMD	14 (17.1)	10 (12.2)		17 (20.7)	7 (8.5)			
REAB1/REAB2	15 (18.3)	20 (24.4)		14 (17.1)	21 (25.6)			
sAML	6 (7.3)	10 (12.2)		6 (7.3)	10 (12.2)			
**Karyotype, n(%)**								
Good	20 (24.4)	15 (18.3)	0.063	21 (25.6)	14 (17.1)	0.077		
Intermediate	10 (12.2)	5 (6.1)		9 (11.0)	6 (7.3)			
Poor	11 (13.4)	21 (25.6)		11 (13.4)	21 (25.6)			
**IPSS, n(%)**								
Low/Int-1	24 (29.3)	8 (9.8)	**0.001**	22 (26.8)	10 (12.2)	**0.024**		
Int-2/high	11 (13.4)	23 (28.0)		13 (15.9)	21 (25.6)			
sAML	6 (7.3)	10 (12.2)		6 (7.3)	10 (12.2)			

miR-320c expression			miR-320d expression			miR-320e expression		
Low (n = 41)	High (n = 41)	P	Low (n = 41)	High (n = 41)	P	Low (n = 41)	High (n = 41)	P
24/17	27/14	0.494	24/17	27/14	0.494	24/17	27/14	0.494
52 (19–78)	49 (20–86)	0.987	53 (19–78)	48 (20–86)	0.444	50 (19–78)	52 (20–86)	0.998
3.5 (0.77–145)	2.88 (0.79–93.58)	0.366	3.27 (0.77–93.58)	3.48 (0.79–145)	0.979	2.95 (0.77–145)	3.5 (0.89–69.9)	0.581
63 (35.5–108.3)	66 (26.3–101)	0.943	64 (35.5–108.3)	62 (26.3–102.6)	0.724	64 (35.5–108.3)	60.1 (26.3–103.3)	0.888
57 (1.92–726.8)	46.3 (4.7–496.1)	0.123	53.1 (1.92–726.8)	48 (4.7–387)	0.613	45.2 (1.92–726.8)	51 (47–496.1)	0.531
4 (0–45)	12 (0–90.5)	**0.003**	4 (0–90.5)	11.2 (0–61)	**0.047**	4 (0–90.5)	8.2 (0–61.5)	0.138

7 (8.5)	0 (0)	**0.006**	5 (6.1)	2 (2.4)	**0.016**	5 (6.1)	2 (2.4)	0.059
15 (18.3)	9 (11)		17 (20.7)	7 (8.5)		16 (19.5)	8 (9.8)	
14 (17.1)	21 (25.6)		11 (13.4)	24 (29.3)		12 (14.6)	23 (28.0)	
5 (6.1)	11 (13.4)		8 (9.8)	8 (9.8)		8 (9.8)	8 (9.8)	
26 (31.7)	9 (11)	**< 0.001**	22 (26.8)	13 (15.9)	**0.024**	21 (25.6)	14 (17.1)	0.176
8 (9.8)	7 (8.5)		9 (11)	6 (7.3)		8 (9.8)	7 (8.5)	
7 (8.5)	25 (30.5)		10 (12.2)	22 (26.8)		12 (14.6)	20 (24.4)	
27 (32.9)	5 (6.1)	**< 0.001**	26 (31.7)	6 (7.3)	**< 0.001**	22 (26.8)	10 (12.2)	**0.012**
9 (11)	25 (30.5)		7 (8.5)	27 (32.9)		11 (13.4)	23 (28.0)	
5 (6.1)	11 (13.4)		8 (9.8)	8 (9.8)		8 (9.8)	8 (9.8)	

MDS group was divided into miR-320 low expression group and miR-320 high expression group according to the median expression level of miR-320 family members. Mann–Whitney’s U test was used to compare the difference of continuous variables between two groups, and Pearson Chi square analysis was used to compare the difference of categorical variables between two groups. *sAML* secondary acute myeloid leukemia (AML transformed from MDS). Bold indicated P < 0.05.

### Diagnostic accuracy of miR-320 family members in MDS

**Figure 3:**
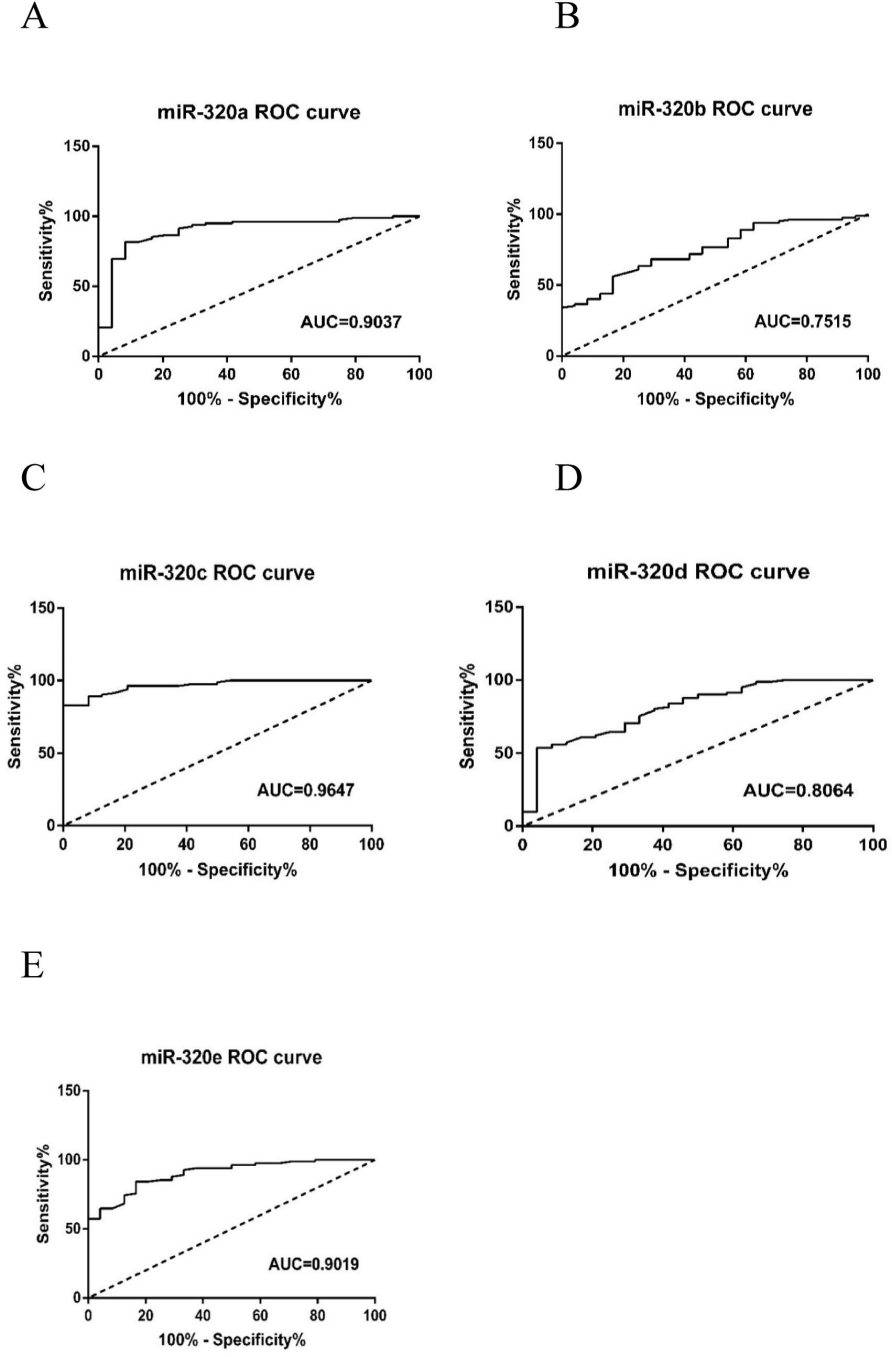
ROC curves for miR-320 family. (**A**) For miR-320a; (**B**) for miR-320b; (**C**) for miR-320c; (**D**) for miR-320d; (**E**) for miR-320e.

We used ROC curve analysis to analyze the diagnostic accuracy of miR-320 family members. The AUC for miR-320 family members was as follows: miR-320a, 0.9037 (95% CI 0.8297–0.9777, P < 0.0001); miR-320b, 0.7515 (95% CI 0.6496–0.8535, P = 0.0002); miR-320c, 0.9647 (95% CI 0.9345–0.9949, P < 0.0001); miR-320d, 0.8064 (95% CI 0.7075–0.9053, P < 0.0001); and miR-320e, 0.9019 (95% CI 0.8407–0.9632, P < 0.0001) (Fig. 3). These results indicated that miR-320 family members could discriminate MDS patients from normal subjects.

### Prognostic implications of miR‑320 family in MDS

**Figure 4:**
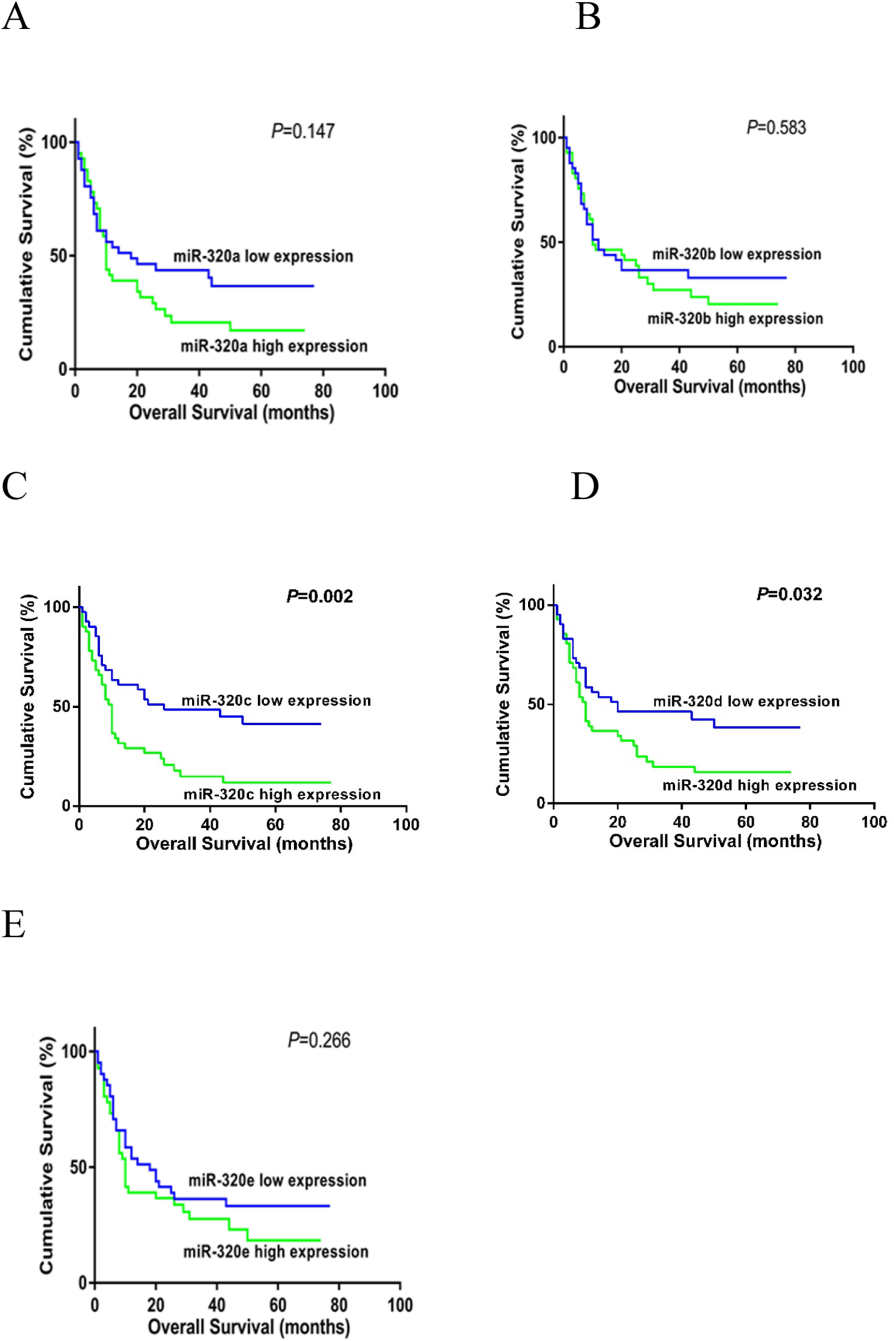
Overall survival of MDS patients with different levels of miR-320 expression. (**A**) For miR-320a; (**B**) for miR-320b; (**C**) for miR-320c; (**D**) for miR-320d; (**E**) for miR-320e. MDS group was divided into miR-320 low expression group and miR-320 high expression group according to the median expression level of miR-320 family members. Kaplan–Meier methods were used to analyze the correlation of miR-320 family expression and OS.

To investigate prognostic implications of miR‑320 family members in MDS, we estimated the relationship between each member of miR-320 family and OS. Regarding on Kaplan–Meier analysis, we discovered that high miR-320c and miR-320d expression were related to shorter OS (P = 0.002 and P = 0.032) (Fig. 4). Additionally, multivariate analysis showed that high expression of miR-320d was an independent prognostic factor for OS (P = 0.020, Table 3).

**Table 3:** Overall survival analyses for MDS patients.

Variables	Univariate analysis		Multivariate analysis	
	HR (95% CI)	P	HR (95% CI)	P
miR-320a	1.447 (0.885–2.481)	0.147	–	–
miR-320b	1.150 (0.693–1.940)	0.583	–	–
miR-320c	2.128 (1.360–3.863)	0.002	–	–
miR-320d	1.725 (1.066–2.999)	0.032	1.434 (1.059–1.942)	0.020
miR-320e	1.327 (0.807–2.265)	0.266	–	–
Age	1.221 (0.721–2.148)	0.446	–	–
Sex	1.171 (0.695–1.974)	0.547	–	–
WBC	0.967 (0.561–1.668)	0.901	–	–
Hb	0.515 (0.181–1.465)	0.334	–	–
Plt	0.782 (0.448–1.366)	0.396	–	–
BM blast	2.987 (1.753–5.088)	< 0.001	2.197 (1.057–4.565)	0.035
WHO type	4.588 (2.189–9.616)	0.015	–	–
Karyotype	2.281 (1.363–3.817)	0.002	–	–
IPSS type	3.075 (1.633–5.791)	< 0.001	1.640 (0.810–3.321)	0.170

Multivariate analysis includes variables with P < 0.05 in univariate analysis.

## Discussion

In present study, we determined the expression of miR-320 family members in the bone marrow of de novo MDS patients. We discovered that miR-320 family members were markedly up-regulated in MDS patients compared with normal control. Additionally, miR-320 family members have been reported to play crucial roles in various cancers. For instance, miR-320a is obviously down-regulated in gastric cancer^15^, breast cancer^16^, lung adenocarcinoma^17^, and osteosarcoma^18^. Furthermore, down-regulation of miR-320a promoted growth and metastasis of cancer cell. However, in pancreatic cancer, miR-320a was evidently up-regulated and enhanced the drug-resistance and proliferation of pancreatic cancer cell^19^. Similarly, miR-320b downregulated in various cancers, including colorectal cancer^20^, nasopharyngeal carcinoma^21^, osteosarcoma^22^ and glioma^23^, indicating that miR-320b might inhibit the progression of these cancers. Moreover, miR-320b was reported to inhibit tumorigenesis by targeting BMI1 in non-small cell lung cancer^24^. Similar with miR-320a/320b, miR-320d acts as an antioncogene in various cancers, such as glioma^25^, prostate cancer^26^, and breast cancer^27^. However, miR-320d was upregulated in pancreatic cancer^28^ and rectal cancer^29^, and high expression of miR-320d was bound up with poor prognosis of rectal cancer. Regarding miR-320c, Iwagami et al. discovered that miR-320c enhanced gemcitabine-resistance by targeting SMARCC1 in pancreatic cancer^30^. Wang et al. demonstrated that miR-320c was significantly elevated in colon cancer and could be regarded as a diagnostic biomarker of early-stage colon cancer^31^. Another study found that miR-320c was decreased in bladder cancer and inhibited tumorous behaviors by targeting CDK6^32^. In regard to miR-320e, Perez-Carbonell et al. demonstrated that miR-320e was significantly elevated in colorectal cancer patients with recurrence compared with colorectal cancer patients without recurrence^33^, indicating that miR-320e expression was associated with the progression of colorectal cancer. Drahos demonstrated that miR-320e was up-regulated in esophageal adenocarcinoma compared to Barrett’s esophagus^34^. In summary, miR-320 family members play anti-tumor roles in most solid tumors except miR-320a, miR-320c and miR-320d, which are increased in pancreatic cancer.

In regard to hematological malignancies, the role of miR-320 family is complex. Circulating miR-320d was confirmed to be markedly increased in AML and has been regarded as a novel diagnostic biomarker for AML^35^. However, miR-320d was significantly decreased in diffuse large B-cell lymphoma (DLBCL)^36^. Low expression of miR-320d was associated with poor prognosis of DLBCL. miR-320a was found to be markedly elevated with a 17.502-fold change in classical Hodgkin lymphoma (cHL) compared with reactive lymphadenopathy^37^. miR-320e was increased with a 3.91-fold change in leukemia stem cells (LSCs) after ponatinib treatment^38^. In multiple myeloma, miR-320 family members acted as tumor suppressors. For instance, miR-320a inhibits tumor growth and increases apoptosis by targeting PBX3 in multiple myeloma^14^. The oncogene EZH2 was able to directly inhibit the expression of miR-320c to promote tumorigenesis in multiple myeloma^39^. Additionally, Mittal et al. reported that miR-320a inhibited erythroid differentiation by targeting SMAR1 in K562 cells (chronic myelocytic leukemia cell lineage)^40^. In our study, the expression of each member of miR-320 family was significantly elevated in MDS patients compared to normal control and showed diagnostic value for MDS. Additionally, high miR-320c and miR-320d expression were associated with poor prognosis of MDS. The exact mechanism and function of miR-320 family members in MDS needed to be study further.

In the present study, we identified miR-320 family members as potential diagnostic and prognostic biomarkers for myelodysplastic syndromes. We demonstrated the that expression of miR-320 family members was up-regulated in MDS, and multivariate analysis showed that high expression of miR-320d was an independent prognostic factor for OS. It was suggested that miR-320 family members might serve as oncogenes in MDS. Targeting miR-320 family members may provide a new strategy for MDS therapy in the future.

## Methods

### Patients

Bone marrow samples were collected from 82 patients with newly diagnosed MDS and AML that transformed from MDS in the Department of Hematology, The First Affiliated Hospital of Guangxi Medical University, China between 2012 and 2017. MDS was diagnosed on the basis of WHO Recommended Criteria (2008). The diagnosis of patients were as follows: 5q-syndrome (n = 3), RA (n = 2), RARS (n = 2), RCMD (n = 24), RAEB-1 (n = 15), RAEB-2 (n = 20) and AML that transformed from MDS (n = 16). The International Prognostic Scoring System (IPSS) was used to calculate the prognostic score of every patient. Clinical parameters of MDS patients were shown in Table 4. All the participants had been given informed consent according to the Declaration of Helsinki. The study was approved by the Human Ethics Committees Review Board at Guangxi Medical University, Nanning, China (Approval Number: 2017(KY-E-098)).

**Table 4:** Clinical parameters of MDS patients and normal controls (qRT-PCR validation).

Features	MDS group	Normal controls	P value
Age (years), median (range)	51 (19–86)	43 (22–61)	0.168
**Sex, n(%)**			
Male	51 (62.2)	15 (62.5)	0.978
Female	31 (37.8)	9 (37.5)	
WBC (× 10^9^/L), median (range)	3.41 (0.77–145)	–	
Hb (g/L), median (range)	63 (26.3–108.3)	–	
PLT (× 10^9^/L), median (range)	49 (1.92–726.8)	–	
BM (%), median (range)	6.5 (0–90.5)	–	

**Karyotype, n(%)**			
Good	35 (42.7)	–	
Intermediate	15 (18.3)	–	
Poor	32 (39)	–	
**WHO type, n(%)**			
RA/RARS	4 (4.9)	–	
5q^-^ syndrome	3 (3.6)	–	
RCMD	24 (29.3)	–	
RAEB-1	15 (18.3)	–	
RAEB-2	20 (24.4)	–	
sAML	16 (19.5)	–	
**IPSS, n(%)**			
Low	3 (3.6)	–	
Int-1	29 (35.4)	–	
Int-2	22 (26.8)	–	
High	12 (14.6)	–	
sAML	16 (19.6)	–	

### RNA isolation

We separated bone marrow mononuclear cells (BM-MNCs) using density gradient centrifugation. Total RNA was isolated from BM-MNCs of 82 patients and 24 controls by TRIzol reagent (Invitrogen, USA) following the manufacturer’s instructions.

### MiRNA microarray analysis

Samples from 8 MDS patients and 6 normal controls were analyzed by the miRCURY LNA Array (v.18.0) (Exiqon) technology^41^. Clinical parameters of 8 MDS patients and 6 normal controls were shown in Table 5. The microarray data was generated as previously reported (PMID: 32629683)^42^. Significant differentially expressed microRNAs between the two groups were defined as P < 0.05 and |logFC|> 1.

**Table 5:** Clinical parameters of MDS patients and normal controls (microarray analysis).

Features	MDS group	Normal controls	P value
Age (years), median (range)	57 (47–73)	48 (46–61)	0.106
**Sex, n(%)**			
Male	5 (62.5)	3 (50)	0.999
Female	3 (37.5)	3 (50)	
WBC (× 10^9^/L), median (range)	2 (0.9–10.0)	–	
Hb (g/L), median (range)	77.6 (33–106.4)	–	
PLT (× 10^9^/L), median (range)	51.7 (38–530.8)	–	
BM (%), median (range)	2 (1–14)	–	

**Karyotype, n(%)**			
Good	4 (42.7)	–	
Intermediate	1 (18.3)	–	
Poor	3 (39)	–	
**WHO type, n(%)**			
RCMD	5 (29.3)	–	
RAEB-2	3 (24.4)	–	
**IPSS, n(%)**			
Int-1	4 (50)	–	
Int-2	2 (25)	–	
High	2 (25)	–	

### qRT-PCR verification

The microarray results were verified by qRT-PCR in 82 patients (MDS and AML that transformed from MDS) and 24 healthy controls. Total RNA was reverse transcribed to cDNA. qRT-PCR was performed as previously reported14. Relative quantification was calculated using the 2^−△△ct^ method and U6 was used for normalization. The primers were as follows: U6 forward: 5′GCTTCGGCAGCACATATACTAAAAT3′ and reverse: 5′CGCTTCACGAATTTGCGTGTCAT3′;miR-320a forward:5′AGCTGGGTTGAGAGGGCG3′ and reverse: 5′GTCGGTGTCGTGGAGTCGTT3′; miR-320b forward: 5′AAGCTGGGTTGAGAGGGCA3′ and reverse: 5′GTCGGTGTCGTGGAGTCGTT3′; miR-320c forward: 5′AAAGGCTGGGTTGAGAGGGT3′ and reverse: 5′GTCGGTGTCGTGGAGTCGTT3′; miR-320d forward: 5′GGAAAAGCTGGGTTGAGAGGA3′ and reverse: 5′GTCGGTGTCGTGGAGTCGTT3′; miR-320e forward: 5′GGGAAAGCTGGGTTGAGAA3′ and reverse: 5′GTCGGTGTCGTGGAGTCGTT3’.

### Statistical analysis

Microarray analysis is based on Student’s t test (8 MDS patients vs. 6 controls), whereas qRT-PCR validation is based on Mann–Whitney’s U test (82 MDS patients vs. 24 controls). Mann–Whitney’s U test and Pearson Chi square test were used to evaluate relationship between miR-320 family members and clinical parameters. Receiver Operator Characteristic (ROC) analysis was used to detect sensitivity, specificity, and the area under the curve (AUC) for miR-320 family members. Kaplan–Meier method used in univariate analysis and Cox proportional hazard regression model in multivariate analysis were used to determine the prognostic value of miR-320 family expression. P < 0.05 indicates statistical significance.
